# Involvement of a dihydrodipicolinate synthase gene (*FaDHDPS1*) in fungal development, pathogenesis and stress responses in *Fusarium asiaticum*

**DOI:** 10.1186/s12866-018-1268-7

**Published:** 2018-10-05

**Authors:** Weichao Ren, Jiting Tao, Dongya Shi, Wenchan Chen, Changjun Chen

**Affiliations:** College of Plant Protection, Nanjing Agricultural University, Nanjing, 210095 Jiangsu China

**Keywords:** *Fusarium asiaticum*, *FaDHDPS1*, Development, Virulence, Stress responses

## Abstract

**Background:**

Dihydrodipicolinate synthase (DHDPS) is an allosteric enzyme, which catalyzes the first unique step of lysine biosynthesis in prokaryotes, higher plants and some fungi. To date, the biological roles of DHDPS in filamentous fungi are poorly understood.

**Results:**

In this study, on the basis of comparative genome resequencing, a DHDPS gene was found to be specific in *Fusarium asiaticum*, named *FaDHDPS1,* which showed high amino acid identity to that of entomopathogenic fungus. Subcellular localization of the FaDHDPS1-GFP fusion protein was mainly concentrated in the cytoplasm of conidia and dispersed in the cytoplasm during conidial germination. To reveal the biological functions, both deletion and complementation mutants of *FaDHDPS1* were generated. The results showed that the *FaDHDPS1* deletion mutant was defective in conidiation, virulence and DON biosynthesis. In addition, deletion of *FaDHDPS1* resulted in tolerance to sodium pyruvate, lysine, low temperature and Congo red.

**Conclusion:**

Results of this study indicate that *FaDHDPS1* plays an important role in the regulation of vegetative differentiation, pathogenesis and adaption to multiple stresses in *F. asiaticum*.

**Electronic supplementary material:**

The online version of this article (10.1186/s12866-018-1268-7) contains supplementary material, which is available to authorized users.

## Background

Fusarium head blight (FHB) is a severely devastating disease of wheat and other cereals caused by a complex of *Fusarium* species and leads to significant economic losses worldwide [1]. In addition to quantitative yield losses, harvested grain sustains qualitative problems of contamination with mycotoxins such as deoxynivalenol (DON), nivalenol (NIV), and zearalenone (ZEN), posing an enormous threat to food and feed production [2]. Due to diversities in climate and cropping systems, different *Fusarium* species are associated with FHB in different wheat growing areas. For Asia, one of the most common *Fusarium* species responsible for FHB is *F. asiaticum* [3, 4]. Despite the high economic impact of FHB on agricultural production, efficient strategies for FHB management have not been well established [5]. Thus, comprehensive understanding of the fundamental biology of this pathogen can provide the basis for sustainable and long-term disease control.

Lysine synthesis starts with the condensation of aspartate-β-semialdehyde (ASA) and pyruvate into dihydrodipicolinic acid in microbes and plants [6]. Dihydrodipicolinate synthase (DHDPS), the enzyme that catalyzes this reaction, is inhibited by the end-product lysine and is therefore thought to have a regulatory control on lysine synthesis [7, 8]. DHDPS has been extensively studied in bacteria and been purified to homogeneity from *Escherichia coli* [9, 10]. Studies of mechanisms in *E. coli* indicated that DHDPS catalyzes the condensation reaction by means of a ping-pong mechanism, that pyruvate binds to a lysine residue in the active site forming a Schiff base, and then the binding of ASA is followed by condensation and release of dihydrodipicolinic acid [9]. Besides lysine synthesis, this pathway produces some intermediate products that are required for the synthesis of cell wall, housekeeping proteins, and virulence factors [11, 12]. Given its importance to pathogenic bacteria and absence in humans, DHDPS is now regarded as a promising antibiotic target [13].

DHDPS has also been studied in various plants, such as tobacco [14], wheat [15], and maize [16]. Structural biology studies of DHDPS protein from tobacco reveals that the plant DHDPS is a homotetramer, which is made up of dimers [8]. The active site and lysine binding site are located at the center and interface of each monomer, respectively. The structure of most DHDPS enzymes from prokaryotes is homotetrameric, apart from *Staphylococcus aureus* and *Pseudomonas aeruginosa*, which being as dimers [17, 18]. Although DHDPS from both bacteria and plants are composed of dimers, they employ a totally different way of dimer assembly [19]. For higher plants, the feedback of DHDPS is also inhibited by the end-product lysine [20]. Thus, Lysine biosynthesis in plants provides a promising target for the design of novel herbicides by lysine inhibition, or improving the nutritional value of crops by increase of lysine production.

For fungi, DHDPS is found not only in plant pathogen, but also in entomogenous fungus, however, to update, the functions of DHDPS is elusive in filamentous fungi, including *Fusarium* spp*.* Comparing the genome sequencing data of *F. asiaticum* wild-type strain 2021 with the *F. graminearum* sequenced strain PH-1, *FaDHDPS1* was unique in *F. asiaticum.* We hypothesized that *FaDHDPS1* played crucial roles in the development and pathogenesis of *F. asiaticum,* thus, main purposes of this study were to characterize the genetic, biological and biochemical functions of *FaDHDPS1*. Our study showed that the *FaDHDPS1* deletion mutant exhibited obviously phenotype changes in vegetative differentiation, virulence and stress tolerance.

## Methods

### Fungal strains and culture conditions

*F. asiaticum* wild-type (WT) strain 2021, which was isolated in wheat field from Zhejiang Province, China, was used as the WT progenitor for sequencing and transformation receipt strain. The WT strain and transformants obtained in this study were cultured at 25 °C on potato dextrose agar (PDA, 200 g potato, 20 g dextrose, 15 g agar and 1 L water) or minimal medium (MM, 10 mM K_2_HPO_4_, 10 mM KH_2_PO_4_, 4 mM (NH_4_)_2_SO_4_, 2.5 mM NaCl, 2 mM MgSO_4_, 0.45 mM CaCl_2_, 9 mM FeSO_4_, 10 mM glucose and 1 L water, pH 6.9) for mycelial growth assays. Mung bean broth (MBL, 30 g of mung beans was boiled in 1 L water for 20 min and filtered through cheesecloth) was used for conidiation assays [21]. Yeast extract peptone dextrose (YEPD, 20 g dextrose, 10 g peptone, 3 g yeast extract and 1 L water) was used for conidial germination.

### Deletion and complementation of *FaDHDPS1* in *F. asiaticum*

The gene replacement construct for deleting *FaDHDPS1* was generated using the method described previously [22]. Briefly, the 5′ and 3′ flanking regions of *FaDHDPS1* were amplified with the primer pairs listed in Additional file 1: Table S1, and then fused with the resistance cassette carrying the hygromycin B resistance gene (*HPH*) and the herpes simplex virus thymidine kinase gene (*HSV-tk*). The resulting PCR products were transformed into the protoplasts of the WT progenitor 2021, as described previously [23]. Hygromycin B and 5-Fluoro-2′-deoxyuridine were added to a final concentration of 100 μg mL^− 1^ and 50 ng mL^− 1^, respectively, for transformants selection. For complementation, the full-length of *FaDHDPS1* was amplified from the genomic DNA of the strain 2021. Before this vector was transformed into the *FaDHDPS1* deletion mutant, *FaDHDPS1* in the vector was sequenced to ensure the flawlessness of the sequence.

Putative gene deletion mutants were identified by PCR assays with the relevant primers (Additional file 1: Table S1), and were further analyzed by the Southern blotting assays (Additional file 2: Figure S1). DNA of each strain was extracted and then digested with the appropriate restriction endonuclease, as indicated in Additional file 2: Figure S1. The probe was labeled with digoxigenin (DIG) using a High Prime DNA Labeling and Detection Starter kit II according to the manufacturer’s instructions (Roche Diagnostics, Mannheim, Germany).

### Mycelial growth and conidiation assays

To test sensitivity of each strain to different stresses, mycelial plugs (5-mm diameter) taken from the edge of a 3-day-old colony were grown in the dark for 3 days at 25 °C in 9-cm diameter Petri plates containing PDA amended with 1.2 M NaCl or 1.2 M KCl (osmotic stress agents), 0.05% (*w*/*v*) SDS (a cell membrane-damaging agent), 0.05% (w/v) Congo red or 5 mM caffeine (cell wall-damaging agents), or 5 mM paraquat or 8 mM H_2_O_2_ (oxidative pressure agents). In addition, mycelial plugs were cultured on MM plates with 50 mM sodium pyruvate as the only carbon source, 2 M lysine as the carbon and nitrogen sources, and MM plates without carbon or nitrogen sources to test the nutrient sources sensitivity. Furthermore, mycelial plugs were cultured on PDA plates in the dark for 20 days at 4 °C to test the sensitivity of each strain to low temperature. Each experiment was repeated three times independently.

For conidiation assays, four 5-mm mycelial plugs from the edge of 3-day-old colony of each strain were inoculated in 30 mL of MBL in a 50-mL flask. After cultivated in a 185-rpm shaker at 25 °C for 5 days, the conidia of each strain were harvested by filtering through cheesecloth and counted by hemacytometer under microscope. Each experiment with three replicates was repeated three times independently.

### RNA extraction and quantitative real-time PCR

To extract total RNA, mycelia of each strain were cultured in glucose yeast extract peptone (GYEP) liquid medium (5% glucose, 0.1% yeast extract and 0.1% peptone) for 7 days shaking incubation in the dark at 28 °C. Total RNA was extracted using the RNAsimple Total RNA Kit (Tiangen Biotech, Beijing, China), and then used for reverse transcription with the HiScript Q Select RT SuperMix for qPCR kit (Vazyme Biotech, Nanjing, China). The expressions of *FaTRI5* and *FaTRI6* were determined by quantitative real-time PCR, and the relative quantification of each transcript was calculated by the 2^-ΔΔCT^ method [24] with the *F. asiaticum* actin gene *FaActin* as the internal control. The experiment was repeated three times independently.

### Plant infection and DON production assays

For plant infection assays, conidia from 5-day-old MBL cultures were harvested and resuspended in sterile distilled water at a concentration of 1 × 10^5^ conidia mL^− 1^. Wheat germs were inoculated with 2 μl conidial suspensions and examined at 10 days post incubation (dpi). Flowering wheat heads of cultivar Zhenmai 5 were drop-inoculated with 10 μl of conidia suspensions at the central spikelet of the spike. 10 μl of sterile distilled water served as controls. Symptomatic spikelets were examined and counted at 15 dpi. For each treatment, 20 samples were inoculated.

For DON production assays, conidia of each strain (1 × 10^5^ conidia mL^− 1^) was added to GYEP liquid medium. After 7 days shaking incubation in the dark at 28 °C, the filtrate was gathered and the mycelia was dried off and weighed. DON was extracted from the filtrate with ethyl acetate, derivatized, mixed with iso-octane and analyzed by gas chromatography-mass spectrometry (GC-MS) according to the method described previously [25]. The experiment was repeated for three times.

### Generation of the FaDHDPS1-GFP fusion cassette

To construct the FaDHDPS1-GFP fusion cassette, *FaDHDPS1* containing the open-reading fragment (without stop codon) and its native promoter region was amplified from the genomic DNA of 2021. The resulting PCR product was cloned into XhoI-digested pYF11 vector by the yeast gap repair approach [26]. Subsequently, the FaDHDPS1-GFP fusion construct was recovered from the yeast transformants using the Yeast Plasmid Kit (Solarbio Biotech, Beijing, China) and then transferred into *E. coli* strain DH5α for propagation. The recombinational plasmid was confirmed by sequencing analysis to contain the in-frame fusion construct and transformed into the *FaDHDPS1* deletion mutant. The resulting G418-resistant transformants were screened by PCR and confirmed by the presence of GFP signals. The primers used are listed in Additional file 1: Table S1.

## Results

### Identification of *FaDHDPS1* in *F. asiaticum*

**Fig. 1:**
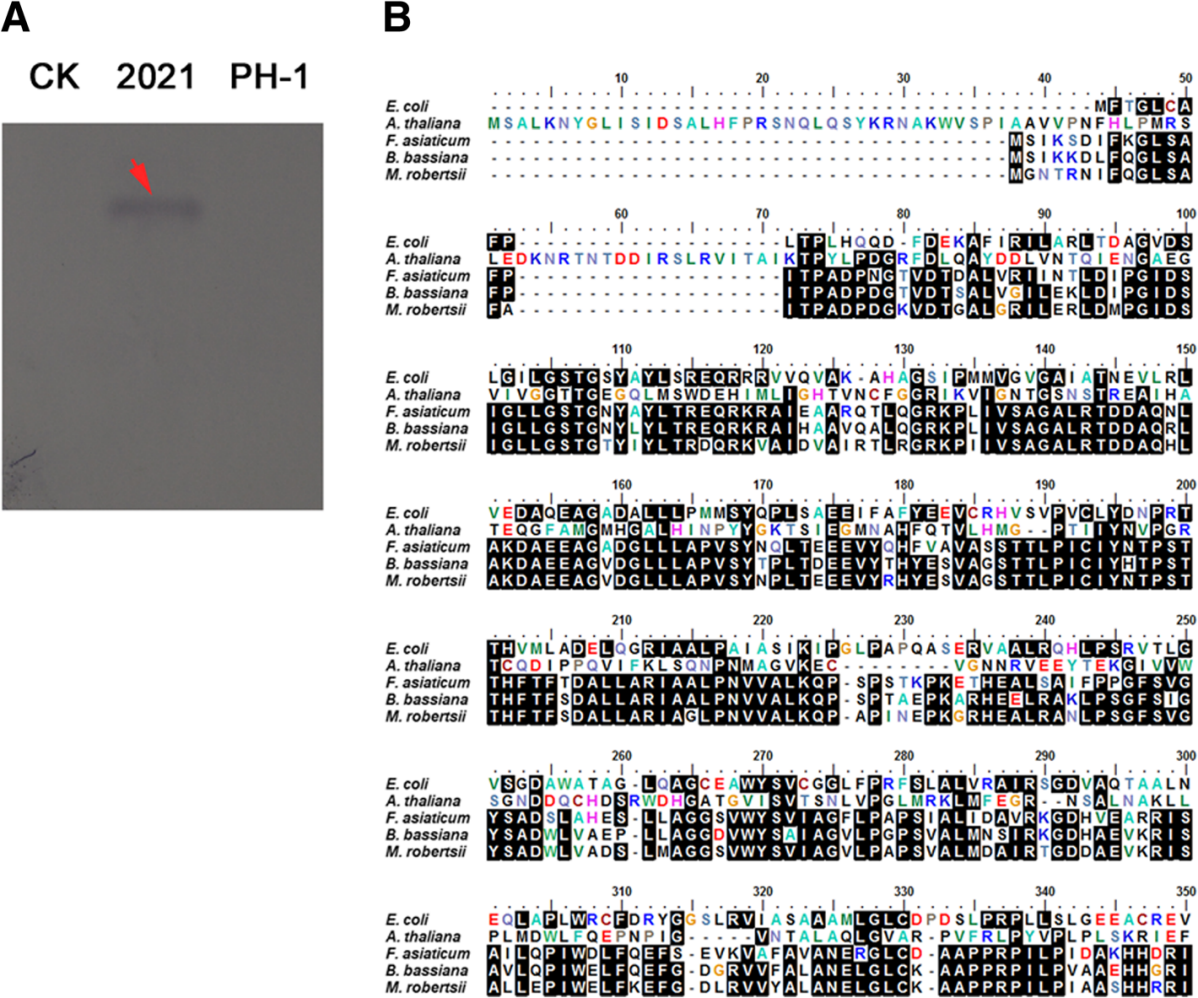
*FaDHDPS1* exists only in *F. asiaticum* among *Fusarium* sp. (**a**) The full-length of *FaDHDPS1* was used as a probe to hybrid the genomic DNA of 2021 and PH-1, and water was used as a negative control. (**b**) Alignment of amino acid sequences of the DHDPS homolog of *F. asiaticum* with those of *Escherichia coli*, *Arabidopsis thaliana*, *Beauveria bassiana* and *Metarhizium robertsii*. The black shading boxshade was used to highlight identical amino acids

The whole genome sequence of *F. asiaticum* wild-type strain 2021was sequenced by the second generation of gene sequencing technology. Compared with the gene sequenced *F. graminearum* strain PH-1, *FaDHDPS1* was found to be a specific gene in *F. asiaticum*. To avoid the sequencing gap mistakes, the full-length of *FaDHDPS1* was labeled as a probe to hybrid the 2021 and PH-1 genomic DNA. As shown in Fig. 1a, the hybrid band was only observed in 2021 indicating that *FaDHDPS1* is unique in 2021.

*FaDHDPS1* was 906 bp in length without any introns, and was predicted to encode a protein with 301 amino acid residues. Blastp searches with *FaDHDPS1* protein sequence as a query showed that the best hit was to entomopathogenic fungi, such as *Beauveria bassiana* and *Metarhizium robertsii* with 77% and 73% amino acid sequence identity, respectively. However, *FaDHDPS1* showed low degree of homology with the counterparts form *E. coli* and *Arabidopsis thaliana* (Fig. 1b).

### Deletion and complementation of *FaDHDPS1* in *F. asiaticum*

To investigate the function of *FaDHDPS1*, target gene deletion mutants of *FaDHDPS1* were generated using a homologous recombination strategy (Additional file 2: Figure S1A). Four putative gene deletion mutants were identified from 16 hygromycin-resistant transformants by PCR analysis with the primer pair P7/P8 (Additional file 1: Table S1). The gene deletion and complementation candidates were further confirmed by Southern blot experiment, as expected, only a 1934 bp band appeared in the gene deletion mutant ΔFaDHDPS1, and only 2253 bp bands appeared in the wild-type progenitor 2021 and complemented strain ΔFaDHDPS1-C (Additional file 2: Figure S1B). The results indicated that ΔFaDHDPS1 was a null mutant resulting from expected recombination events at the *FaDHDPS1* locus, and ΔFaDHDPS1 was complemented in situ by a single copy of *FaDHDPS1*.

### Involvement of *FaDHDPS1* in conidial production and germination

**Fig. 2:**
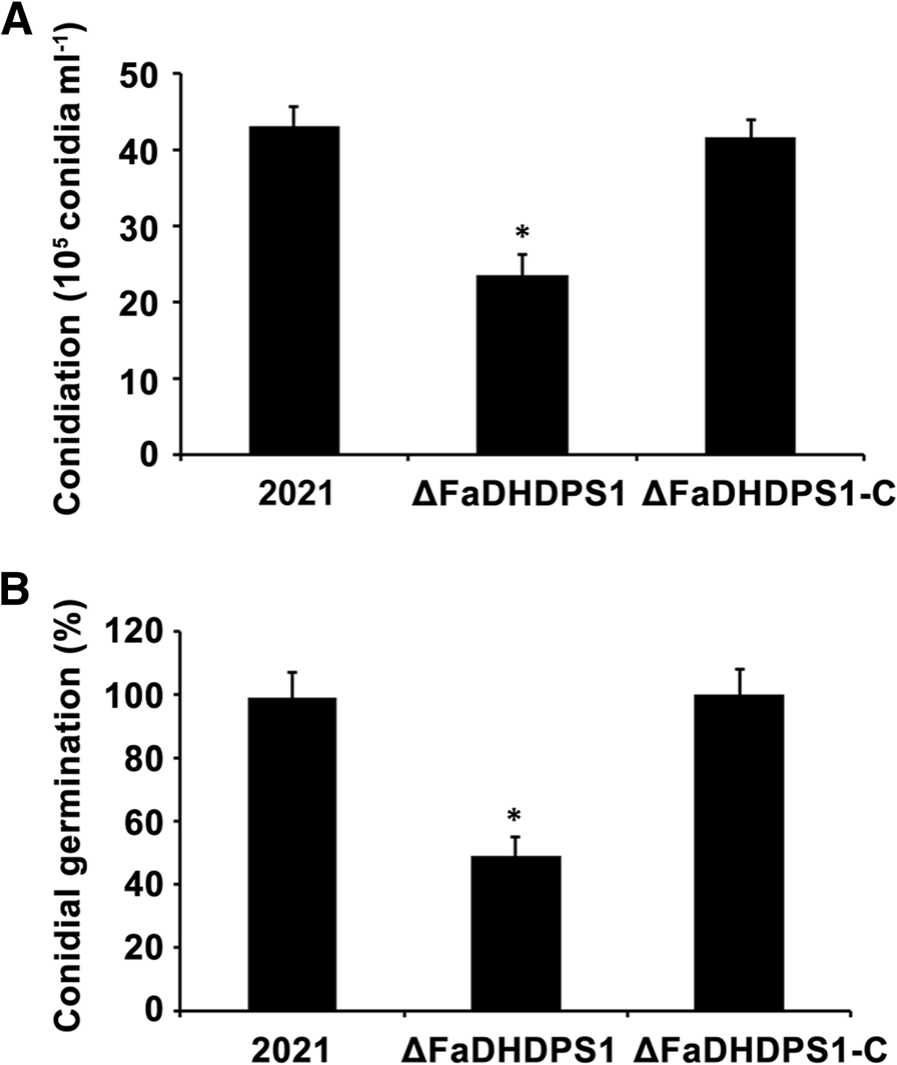
Impacts of *FaDHDPS1* on conidial production and germination. (**a**) Conidia were accounted after incubation of the wild-type strain 2021, the *FaDHDPS1* deletion mutant ΔFaDHDPS1 and complemented strain ΔFaDHDPS1-C in mung bean liquid (MBL) medium for 5 days in a shaker. Bars denote the standard errors from three experiments. Asterisks indicate statistically significant differences (*P* < 0.05). (**b**) Percentages of germinated conidia of 2021, ΔFaDHDPS1 and ΔFaDHDPS1-C after incubated in YEPD medium for 6 h. Bars denote standard errors from three experiments. Asterisks indicate statistically significant differences (*P* < 0.05)

The *FaDHDPS1* deletion mutant ΔFaDHDPS1 grew slightly slower than the wild-type progenitor 2021 and the complemented strain ΔFaDHDPS1-C on PDA and MM medium, but the difference is not significant. Since asexual reproduction is important for the disease spread of *F. asiaticum*, the conidiation capacity was tested. After incubated in MBL medium for 5 days, the number of conidia produced by ΔFaDHDPS1 was 45% less than that of the wild-type progenitor 2021 (Fig. 2a). In addition, conidial germination was tested, after 6 h incubation in YEPD medium at 25 °C, the germination rate was nearly 100% in the strain 2021 but only 48% in ΔFaDHDPS1 (Fig. 2b). These results indicate that *FaDHDPS1* is essential for growth, conidiogenesis and germination in *F. asiaticum*.

### Effects of *FaDHDPS1* deletion on sensitivity to nutrient sources

**Fig. 3:**
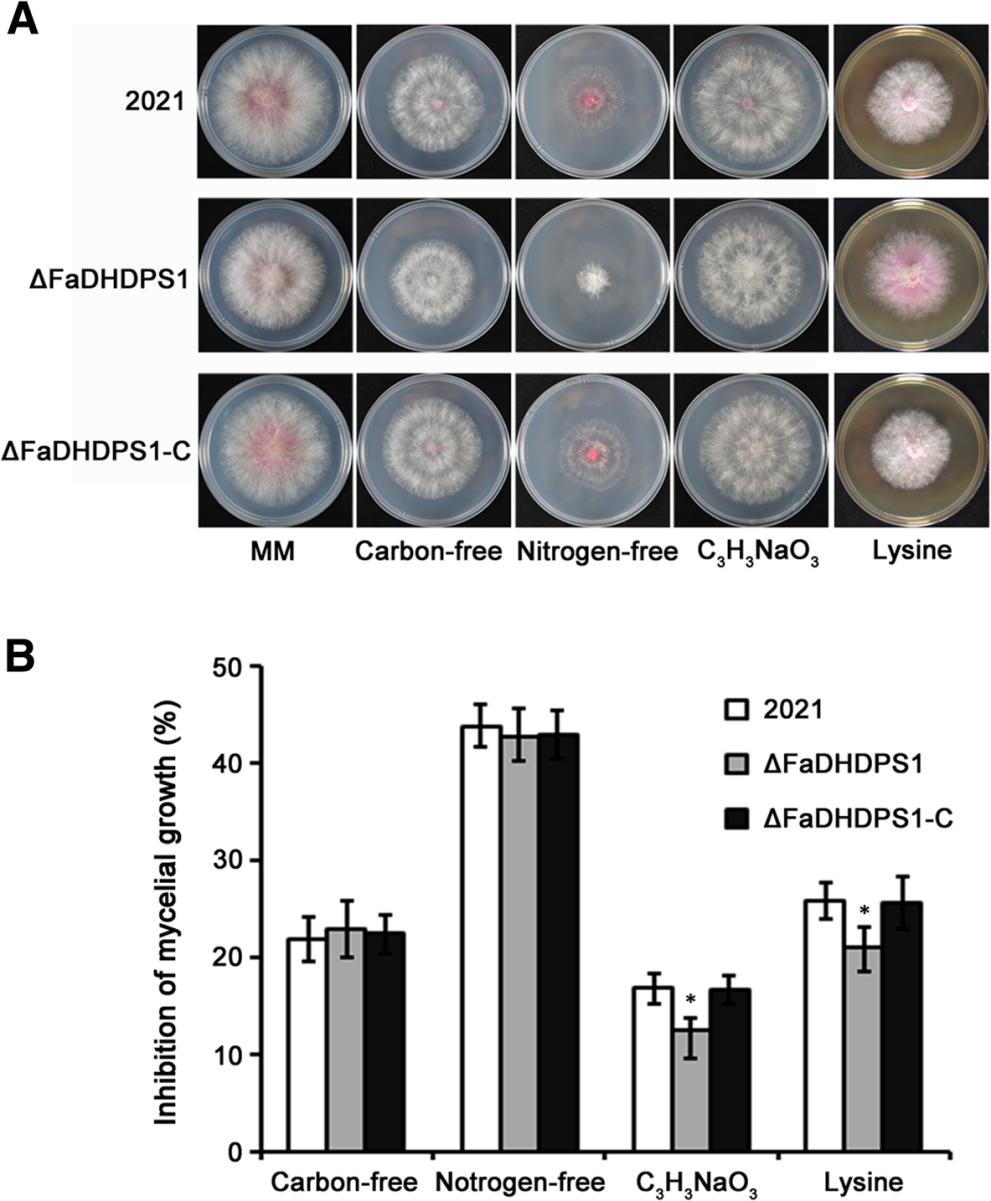
Sensitivity of the wild-type strain 2021, *FaDHDPS1* deletion mutant ΔFaDHDPS1 and complemented strain ΔFaDHDPS1-C to carbon-free,nitrogen-free,C_3_H_3_NaO_3_ and Lysine on MM (**a**) Colony morphology of 2021, ΔFaDHDPS1 and ΔFaDHDPS1-C, the images were photographed after 4 days at 25 °C. (**b**) Inhibition rate of mycelial growth on each stress indicated in figure. Bars denote the standard errors from three experiments. Asterisks indicate statistically significant differences (*P* < 0.05)

DHDPS catalyzes the condensation of ASA and pyruvate to generate dihydrodipicolinic acid in lysine biosynthesis pathway of bacteria and plants [27]. We, therefore, tested the sensitivity of ΔFaDHDPS1 to carbon or nitrogen source on MM medium. The mycelial growth rates of ΔFaDHDPS1 was slightly slower than those of 2021 and ΔFaDHDPS1-C on MM without carbon or nitrogen source, while its aerial hyphae and pigment decreased significantly on MM without nitrogen source (Fig. 3a). Notably, the inhibition of mycelial growth of ΔFaDHDPS1 was lower on MM amended with 50 mM sodium pyruvate or 2 M lysine than that of wild-type 2021 (Fig. 3b). These results suggested that *FaDHDPS1* is involved in the regulation of material metabolism in *F. asiaticum*.

### Effects of *FaDHDPS1* deletion on sensitivity to environmental stresses

**Fig. 4:**
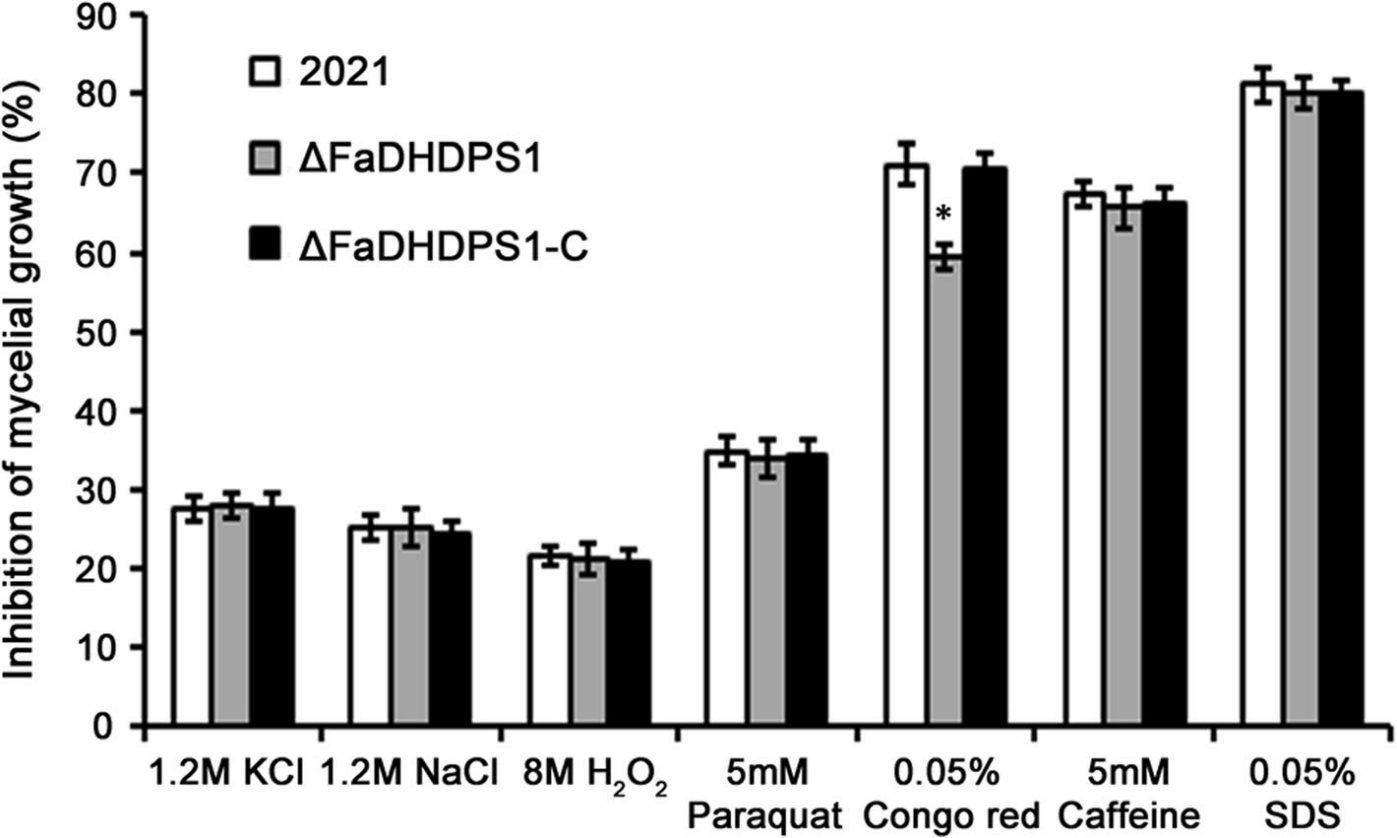
Comparison of sensitivity of 2021, ΔFaDHDPS1 and ΔFaDHDPS1-C to various environmental stresses. Inhibition of mycelial growth was examined after each strain had been incubated for 3 days on PDA amended with the osmotic stress NaCl or KCl, the oxidative stress H O or paraquat and the cell wall and cytoplasm membrane stress caffeine, Congo red or SDS. Bars denote the standard errors from three experiments. Asterisks indicate statistically significant differences (*P* < 0.05)

To assess the biological fitness of ΔFaDHDPS1, the sensitivity to osmotic stresses KCl and NaCl, oxidative stresses H_2_O_2_ and paraquat, and cell wall damaging agents Congo red, caffeine and SDS was examined. The results showed that deletion of *FaDHDPS1* did not alter the sensitivity of ΔFaDHDPS1 to stresses generated by the above substances, except for Congo red (Fig. 4), namely, the sensitivity of ΔFaDHDPS1 to Congo red was significantly decreased compared with wild-type and complemented strains.

### Effects of *FaDHDPS1* deletion on sensitivity to low temperature

**Fig. 5:**
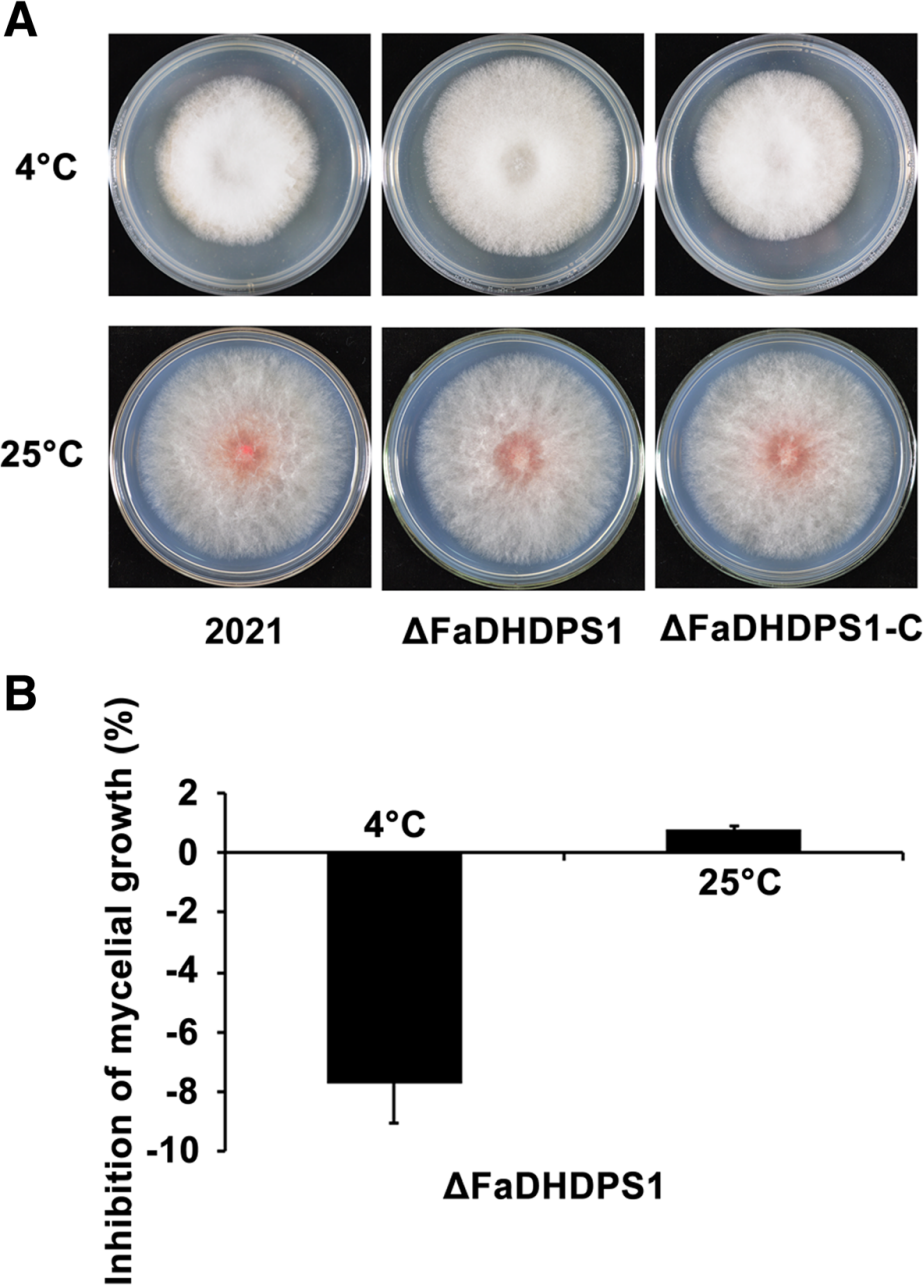
Sensitivity determination of the *FaDHDPS1* deletion mutant to temperature. (**a**) Colony growth of the wild-type 2021, the *FaDHDPS1* deletion mutant △FaDHDPS1 and complemented strain FaDHDPS1-C on PDA at 4 °C and 25 °C. Colonies were photographed after 20 days and 3 days for 4 °C and 25 °C, respectively. (**b**) The inhibition rate of mycelial growth on PDA at 4 °C and 25 °C. Values are the means ± standard error (SE) of three repeated experiments

The occurrence of FHB is strongly determined by weather conditions, and temperature is a key factor to affect the variety distribution of *Fusarium* species [28], we, therefore, were interested in the effect of *FaDHDPS1* on thermosensitivity. As shown in Fig. 5, ΔFaDHDPS1 grew slightly slower than the wild-type strain 2021 on PDA at 25 °C, whereas ΔFaDHDPS1 grew significantly faster than 2021 in low temperature 4 °C. These results suggest that *FaDHDPS1* plays an important role in cold stress adaption.

### Requirement of *FaDHDPS1* in the pathogenicity of *F. asiaticum*

**Fig. 6:**
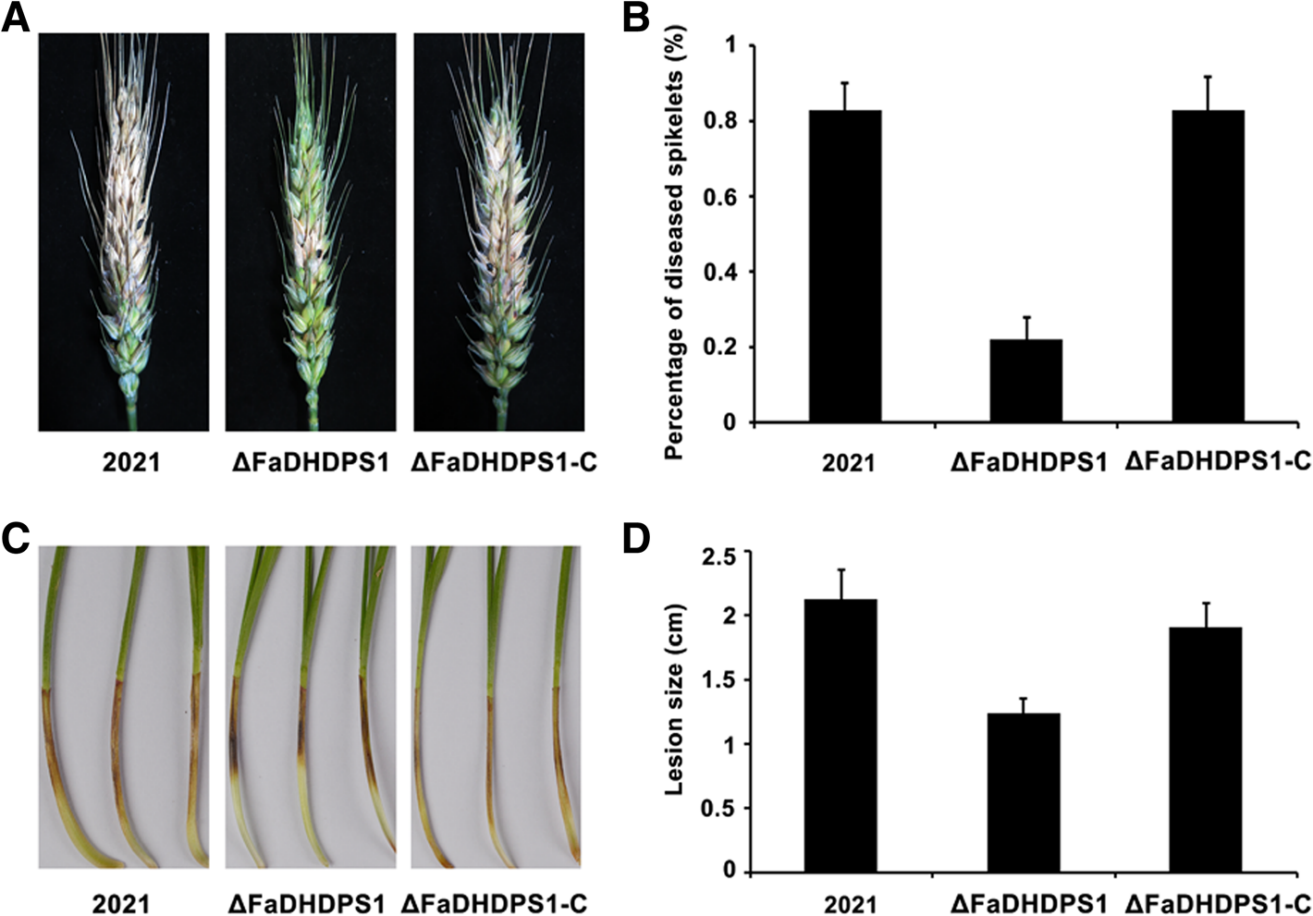
Virulence of the *FaDHDPS1* deletion mutant. (**a**) Wheat germs were incubated with conidial suspensions of 2021, ΔFaDHDPS1 and ΔFaDHDPS1-C and examined after 10 days post incubation (dpi). (**b**) The lengths of diseased lesions of wheat germs were measures 10 dpi. (**c**) Flowering wheat heads were incubated with the conidia of each strain and photographed at 15 dpi. (**d**) Percentage of diseased spikes on inoculated wheat heads. Values are the means ± standard error (SE) of three repeated experiments

To determine whether *FaDHDPS1* is related to pathogenicity in *F. asiaticum*, the virulence of Δ*FaDHDPS1* was evaluated by point inoculation of conidial suspension on flowering wheat heads. 15 days after inoculation, scab symptoms caused by ΔFaDHDPS1 were restricted near the inoculated spikelets. However, the wild-type and the complemented strains caused severe and typical scab symptoms (Fig. 6a and b), suggesting that *FaDHDPS1* is required for full virulence of *F. asiaticum.*

Pathogenicity was also assessed on wheat germs, the ΔFaDHDPS1 strain caused significantly smaller lesions than the wild-type and the complemented strains (Fig. 6c and d). These results indicate that *FaDHDPS1* is required for full virulence of *F. asiaticum*.

### Role of *FaDHDPS1* in the regulation of deoxynivalenol (DON) biosynthesis

**Fig. 7:**
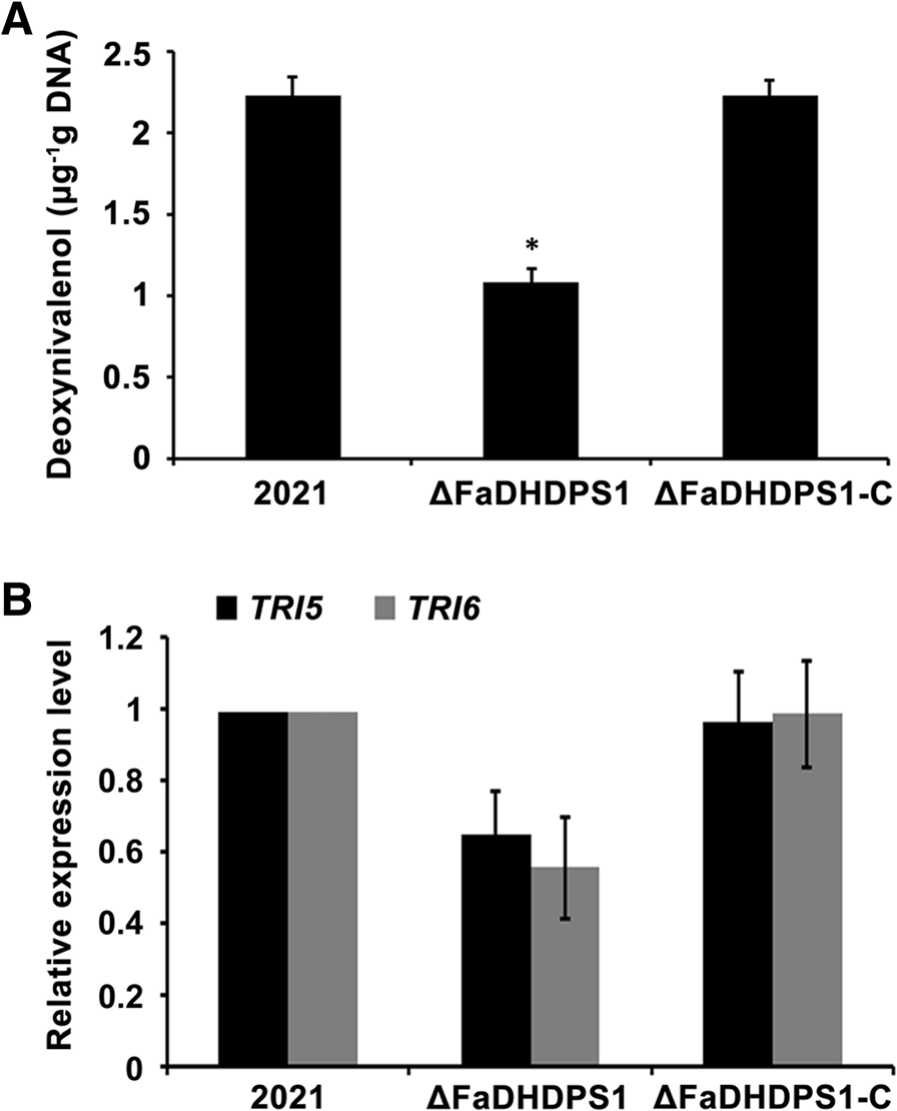
Deoxynivalenol (DON) production and the expression of DON biosynthesis genes. (**a**) DON production by each strain in GYEP medium was examined by gas chromatography. Line bars in each column denote standard errors of three repeated experiments. Asterisks indicate statistically significant differences (*P* < 0.05). (**b**) Relative expression levels of *TRI5* and *TRI6* were measured by quantitative real-time PCR. Line bars in each column denote standard errors of three repeated experiments

Previous studies have shown that DON is a prerequisite for primary infection of *F. graminearum* [29]. Since the virulence of ΔFaDHDPS1 was significantly reduced, we examined the impact of *FaDHDPS1* on DON biosynthesis. The amount of DON produced by ΔFaDHDPS1 was one-half less than the wild-type and complemented strains (Fig. 7a). To get more clues, we detected the transcriptional levels of two trichothecene biosynthesis genes, *TRI5* and *TRI6*, by quantitative real-time PCR. Compared with the wild-type and complemented strains, the expression levels of *TRI5* and *TRI6* in ΔFaDHDPS1 reduced by 36% and 45%, respectively (Fig. 7b). These results indicate that *FaDHDPS1* plays a critical role in DON biosynthesis in *F. asiaticum*.

### Subcellular localization of FaDHDPS1-GFP fusion protein

**Fig. 8:**
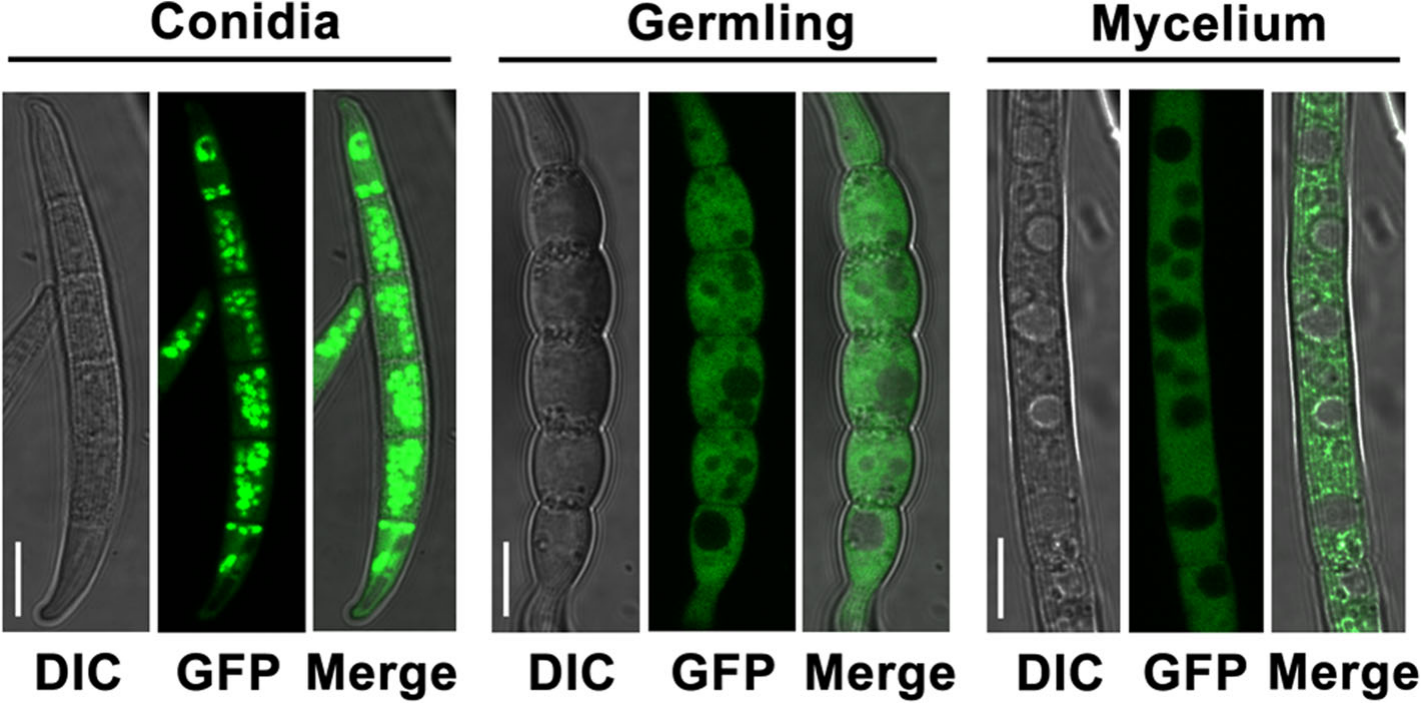
Subcellular localization of *FaDHDPS1* in various developmental stages of *Fusarium asiaticum*. A FaDHDPS1-GFP fusion construct under native promoter was introduced into the *FaDHDPS1* deletion mutant. The fluorescence signals were detected in conidia, germlings and mycelia. Bar = 10 μm

To further explore the function of *FaDHDPS1*, we generated FaDHDPS1-GFP fusion construct and transformed it into ΔFaDHDPS1. The expression of FaDHDPS1-GFP fusion protein was detected by Western blotting using anti-GFP antibody (Additional file 3: Figure S2). The phenotypic defects of ΔFaDHDPS1 were restored, which indicated that the FaDHDPS1-GFP fusion protein was fully functional (data not shown). FaDHDPS1-GFP was mainly localized in the cytoplasm of both conidia and hyphae (Fig. 8). Further examination showed that the GFP fluorescence which concentrated in conidia diffused in cytoplasm of germinated conidia and growing hyphae (Fig. 8) suggesting that *FaDHDPS1* is crucial for conidial germination, which is consistent with the germination delay of ΔFaDHDPS1.

## Discussion

Lysine is one of the most essential amino acids that cannot be synthesized in human and animal [6]. In prokaryotes and higher plants, DHDPS catalyzes the first unique step of lysine biosynthesis [8]. The potential of lysine biosynthesis as an anti-microbial target is increased because diaminopimelate, a precursor in the mesodiaminopimelic acid pathway to lysine, is essential in bacterial cell-wall development [30]. Lysine biosynthesis has also attracted the attention of biotechnologists, since lysine is nutritionally limiting for humans and livestock in most cereal crops and feeds [31], and understanding the regulatory mechanisms of this pathway is thus critical for the generation of ‘high-lysine’ plants. However, up to now, DHDPS has not been functionally characterized in filamentous fungi. In the present study, a DHDPS gene was identified and characterized in *F. asiaticum*, a severe fungal pathogen of cereal crops. Our data showed that *FaDHDPS1* is required for fungal development, virulence and stress responses in *F. asiaticum*. To our knowledge, this is the first report about biological functions of DHDPS in filamentous fungi*.*

Homologous sequence alignment via Blast showed no DHDPS counterparts exist in other *Fusarium* species, and the *FaDHDPS1* coding amino acids showed high homology to that of entomopathogenic fungi, but low homology to that of bacteria and plants, indicating that *DHDPS* may perform a specific role in kingdoms.

The deletion of *FaDHDPS1* in *F. asiaticum* was viable. Previous studies reported that DHDPS is related to growth in *Streptococcus pneumonia*, a Gram-positive bacterium [32], and lacking the DHDPS gene in *S. pneumoniae* is unable to grow. In this study, the *FaDHDPS1* deletion mutant showed slightly reduced mycelial growth rate on PDA and MM medium, even in MM without carbon or nitrogen sources, while the obvious reduction of aerial hyphae and pigment in the *FaDHDPS1* deletion mutant was found when cultured on MM without nitrogen source. The results indicated that *FaDHDPS1* is not only associated with the assimilation of carbon and nitrogen sources, but also involved in pigment biosynthesis in *F. asiaticum*. Although the inhibition ratio of ΔFaDHDPS1 mutant is lower on MM with sodium pyruvate and lysine respectively, its colony diameter was not significantly different from the parental strain 2021 and complemented strain. The specific function of DHDPS in the lysine biosynthesis pathway of *F. asiaticum* needs further research.

DHDPS delivers the direct precursor of dipicolinic acid which plays a key role in the bacterial sporulation process [33]. In this study, the ΔFaDHDPS1 mutant presented serious defects in conidiation, which is consistent with the crucial role of DHDPS in bacteria [32]. In addition, the conidial germination is severely delayed in ΔFaDHDPS1 mutant compared with that in wild-type strain. Furthermore, the dynamic change of the FaDHDPS1-GFP fusion protein also demonstrated that *FaDHDPS1* is required for conidiation. Thus, *FaDHDPS1* may be essential for providing materials for conidial formation and germination.

*FaDHDPS1* was lysine-insensitive in *F. asiaticum*. The sensitivity of DHDPS to lysine differs widely. DHDPS from plants is strongly inhibited by lysine [34], while DHDPS from gram-negative bacteria such as *E. coli* [35] is less strongly inhibited by lysine, and most gram-positive bacteria, such as *Bacillus subtilis* [36] and *Corynebacterium glutamicum* [37], DHDPS is not inhibited by lysine at all. Therefore, *FaDHDPS1* was similar to the DHDPS from most gram-positive bacteria.

*FaDHDPS1* may be involved in the regional distribution of *F. asiaticum*. The response of ΔFaDHDPS1 mutant to low temperature decreased significantly when compared with the parental strain 2021, and this may be explained at a certain extend that the population distribution of *F. asiaticum* in China, where FHB caused by *F. asiaticum* mainly dominates in the south of the Yangtze river, in which the annual average temperature is greater than 15 degrees, whereas *F. graminearum* s.st. mainly thrives in the north of the Huanghe river, where the annual average temperature is less than 15 degrees [38].

The previous study reported that DHDPS is required for virulence in human pathogen *Neisseria meningitides* [39]. In this study, the *FaDHDPS1* deletion mutant showed considerably attenuated virulence on both germ and spike of wheat. In addition, the *FaDHDPS1* deletion mutant exhibited significantly reduced deoxynivalenol production in vitro which is consistent with the inability of ΔFaDHDPS1 mutant to infect plant. To further confirm this result, the expression level of two trichothecene biosynthesis genes *TRI5* and *TRI6* were detected by quantitative real-time PCR. The expression levels of these two genes were considerably decreased in the ΔFaDHDPS1 mutant. The deletion of *FaDHDPS1* might reduce trichothecene biosynthesis through an indirect effect on secondary metabolism, which is consistent with the conclusion that knockouts of most genes reduce toxin biosynthesis and attenuate virulence [40] (Kazan et al., 2012).

## Conclusion

Our data provided strong evidence that *FaDHDPS1* is involved in diverse cellular processes in filamentous fungi *F. asiaticum*, including conidiation, conidial germination, cell wall pressure response, low temperature tolerance, carbon/nitrogen sources utilization, secondary metabolism and virulence. The future work will focus on the role of *FaDHDPS1* in lysine biosynthesis.

## Additional files

Additional file 1: **Table S1.** Oligonucleotide primers used in this study. (DOCX 18 kb)

Additional file 2: **Figure S1.** Generation and identification of *Fusarium asiaticum FaDHDPS1* gene deletion mutants. (A) Gene replacement and complementation strategy for *FaDHDPS1*. The gene replacement cassette *HPH-HSV-tk* contains the hygromycin resistance gene and the herpes simplex virus thymidine kinase gene. Primer binding sites are indicated by arrows (see Additional file 1: Table S1 for the primer sequences). (B) Southern blot hybridization analysis of 2021, ΔFaDHDPS1 and ΔFaDHDPS1-C using the upstream fragment of *FsDHDPS1* as a probe, and genomic DNA were digested with SphI. (TIF 1328 kb)

Additional file 3: **Figure S2.** Western blotting assays of the expression of FaDHDPS1-GFP fusion protein. The total proteins from ΔFaDHDPS1::FaDHDPS1-GFP strains were detected with the anti-GFP antibody. The wild-type strain 2021 and ΔFaDHDPS1::GFP were used as controls. (TIF 1911 kb)

## Abbreviations

ASA: Aspartate-β-semialdehyde
DHDPS: Dihydrodipicolinate synthase
DON: Deoxynivalenol
dpi: days post incubation
FHB: Fusarium head blight
GC-MS: Gas chromatography-mass spectrometry
GYEP: Glucose yeast extract peptone
*HPH*: Hygromycin B resistance gene
*HSV-tk*: Herpes simplex virus thymidine kinase gene
MBL: Mung bean broth
MM: Minimal medium
NIV: Nivalenol
PDA: Potato dextrose agar
RGI: Mycelial growth inhibition
WT: Wild-type
ZEN: Zearalenone
