# Supplementary material for: Involvement of a dihydrodipicolinate synthase gene (FaDHDPS1) in fungal development, pathogenesis and stress responses in Fusarium asiaticum
Source: BMC Microbiol. 2018 Oct 5;18:128. doi: 10.1186/s12866-018-1268-7 (PMC6173861; doi:10.1186/s12866-018-1268-7)
Supplement: Supplementary file 1 — Table S1. Oligonucleotide primers used in this study. (DOCX 18 kb) [file 12866_2018_1268_MOESM1_ESM.docx]

**Table S1.** Oligonucleotide primers used in this study.

| Primer | Sequence (5’-3’) | Relevant Characteristics |
| --- | --- | --- |
| P1 | CAAGCGGCTGTTGTGTAG | Primers to amplify *FaDHDPS1* upstream fragment for construction of the gene deletion vector |
| P2 | CTTCAATATCATCTTCTGTGT TGATGGAGATGCTATAAAG |  |
|  |  |  |
| P3 | AGGAGACAATACCGGAAGGA  ACCTAAGTTTCAAGGTAGCCG | Primers to amplify *FaDHDPS1* downstream fragment for construction of the gene deletion vector |
| P4 | AGGCATCAGGAGGCATTTC |  |
|  |  |  |
| P5 | CATCTACTTCGATGAAATC | Primers to amplify the *FaDHDPS1* gene deletion vector |
| P6 | GGACTCACTACTAATCCTC |  |
|  |  |  |
| P7 | CAATGAACTTACCAACTG | PCR primers for identification of *FaDHDPS1* deletion transformants |
| P8 | GTCTCGGTCTACCTACATC |  |
|  |  |  |
| P9 | ACTCACTATAGGGCGAATTGGG  TACTCAAATTGGTTGCCTCTTG  AACACTCTTACC | PCR primers to amplify *FaDHDPS1* fragment used for construction of the FaDHDPS1-GFP vector under native promoter |
| P10 | CACCACCCCGGTGAACAGCTCC  TCGCCCTTGCTCACAAAGCTTT  TATTGATGTCTG |  |
|  |  |  |
| P11 | GCACCATCAATTGCGCTGAT | PCR primers for the identification of the in-frame FaDHDPS1-GFP fusion vector from yeast |
| P12 | GACACGCTGAACTTGTGGCCGTT |  |
|  |  |  |
| P13 | AGCAGATGGTTGCTGTCTTCT | PCR primers for amplification of the *FaTRI5* gene in quantitative real-time PCR assays |
| P14 | TTCTGAGCCTCCTTCACATCG |  |
|  |  |  |
| P15 | AAATGCCCATTCCCTAGTTG | PCR primers for amplification of the *FaTRI6* gene in quantitative real-time PCR assays |
| P16 | ATCTCGCATGTTATCCACCCT |  |
|  |  |  |
| P17 | ATCCACGTCACCACTTTCAA | PCR primers for amplification of the *FaActin* gene in quantitative real-time PCR assays |
| P18 | TGCTTGGAGATCCACTTTG |  |
|  |  |  |
| P19 | CCAGTCCAAATCGTAGAGTC | Primers to amplify *FaDHDPS1* upstream fragment used as the probe for Southern blot analysis |
| P20 | GTGAGCTCGGAGCTCGGATC |  |
|  |  |  |
