# Supplementary figures and images for: Involvement of a dihydrodipicolinate synthase gene (FaDHDPS1) in fungal development, pathogenesis and stress responses in Fusarium asiaticum
Source: BMC Microbiol. 2018 Oct 5;18:128. doi: 10.1186/s12866-018-1268-7 (PMC6173861; doi:10.1186/s12866-018-1268-7)

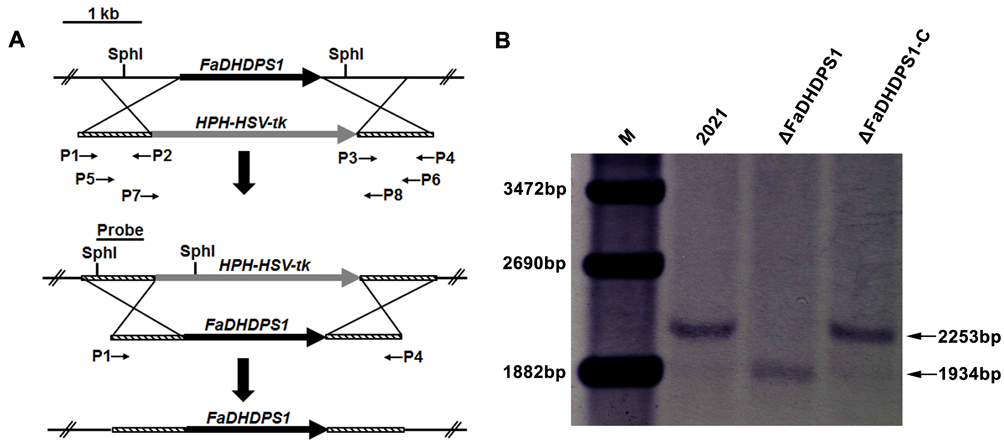

Supplement: Supplementary file 2 — Figure S1. Generation and identification of Fusarium asiaticum FaDHDPS1 gene deletion mutants. (A) Gene replacement and complementation strategy for FaDHDPS1. The gene replacement cassette HPH-HSV-tk contains the hygromycin resistance gene and the herpes simplex virus thymidine kinase gene. Primer binding sites are indicated by arrows (see Additional file 1: Table S1 for the primer sequences). (B) Southern blot hybridization analysis of 2021, ΔFaDHDPS1 and ΔFaDHDPS1-C using the upstream fragment of FsDHDPS1 as a probe, and genomic DNA were digested with SphI. (TIF 1328 kb) [file 12866_2018_1268_MOESM2_ESM.tif]

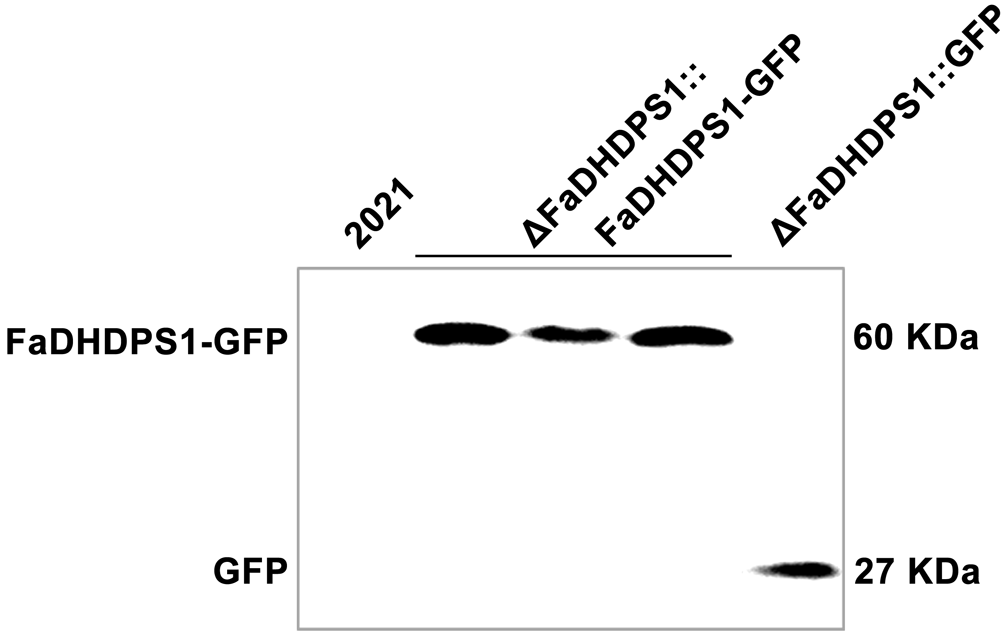

Supplement: Supplementary file 3 — Figure S2. Western blotting assays of the expression of FaDHDPS1-GFP fusion protein. The total proteins from ΔFaDHDPS1::FaDHDPS1-GFP strains were detected with the anti-GFP antibody. The wild-type strain 2021 and ΔFaDHDPS1::GFP were used as controls. (TIF 1911 kb) [file 12866_2018_1268_MOESM3_ESM.tif]
